# A Study of the Variability in Contact Resistive Random Access Memory by Stochastic Vacancy Model

**DOI:** 10.1186/s11671-018-2619-x

**Published:** 2018-07-16

**Authors:** Yun-Feng Kao, Wei Cheng Zhuang, Chrong-Jung Lin, Ya-Chin King

**Affiliations:** Microelectronics Laboratory, Institute of Electronics Engineering, National Tsing Hua University, Hsinchu, 300 Taiwan

**Keywords:** RRAM, Variability, Stochastic model, Monte Carlo simulation, Trap-assisted tunneling

## Abstract

Variability in resistive random access memory cell has been one of the critical challenges for the development of high-density RRAM arrays. While the sources of variability during resistive switching vary for different transition metal oxide films, the stochastic oxygen vacancy generation/recombination is generally believed to be the dominant cause. Through analyzing experimental data, a stochastic model which links the subsequent switching characteristics with its initial states of contact RRAM cells is established. By combining a conduction network model and the trap-assisted tunneling mechanism, the impacts of concentration and distribution of intrinsic oxygen vacancies in RRAM dielectric film are demonstrated with Monte Carlo Simulation. The measurement data on contact RRAM arrays agree well with characteristics projected by the model based on the presence of randomly distributed intrinsic vacancies. A strong correlation between forming characteristics and initial states is verified, which links forming behaviors to preforming oxygen vacancies. This study provides a comprehensive understanding of variability sources in contact RRAM devices and a reset training scheme to reduce the variability behavior in the subsequent RRAM states.

## Background

Resistive random access memory (RRAM) has been regarded as a promising nonvolatile data storage solution, as a result of its desirable features, such as low power, high P/E speed, and superior compatibility with CMOS logic process [1–4]. However, there are still many obstacles to be overcome to easily implement RRAM memory arrays in current state-of-the-art CMOS circuits [5, 6]. One of the key challenges in sizable RRAM array is found in the variation existing between and within cells [7–10]. Many models and simulations have been proposed to describe the stochastic generation/recombination process of oxygen vacancy (Vo-) in transition metal oxide (TMO) film [11–14]. Kim and Brivio proposed random circuit breaker network models to emulate the typical electric characteristics of unipolar and bipolar RRAM, respectively [11, 12]. However, the resistors in these studies were all set to be constant without considering electron transportation in RRAM film. Besides, because presented models discuss stochastic processes of RRAM from a single device level instead of statistical analysis, the variability of RRAM behavior in an array are not well addressed and discussed in previous work [11–14]. Furthermore, the presence of defects in dielectric film during fabrication has been studied extensively for many years [15, 16], but its impact to resistive switching characteristics in RRAM still needs to be comprehensively analyzed for the technology to be applied in sizable memory macros. To investigate the effect of intrinsic Vo- distribution on the RRAM characteristics, a resistor network modeled on the trap-assisted tunneling mechanism is built for further statistical analysis of the variation and during operations in this study [11–14, 17]. Besides, stochastic generation process of Vo- is simulated by Monte Carlo method to establish the correlation between the RRAM in its initial states and the following forming characteristics [18–20]. The strong correlation between intrinsic Vo- and forming voltage is established by verifying the simulation result with measured data on contact RRAM arrays [21]. Finally, different types of conductive filament (CF) generated and resistance state variation after forming operations as a result of the intrinsic Vo- distribution are projected and investigated comprehensively. In addition, a solution for relieving the impact of preforming Vo- on variability is proposed and demonstrated in this study.

## Methods

**Fig. 1:**
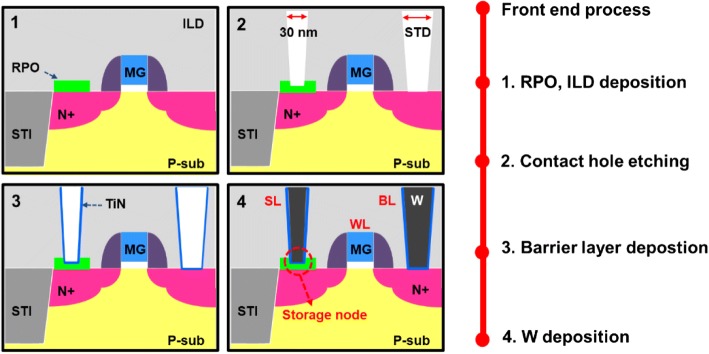
Process flow of contact RRAM on a 28-nm high-k metal gate CMOS logic process platform. Smaller contact size for CRRAM is designed to control etching thickness to form functional resistive switching layer

**Fig. 2:**
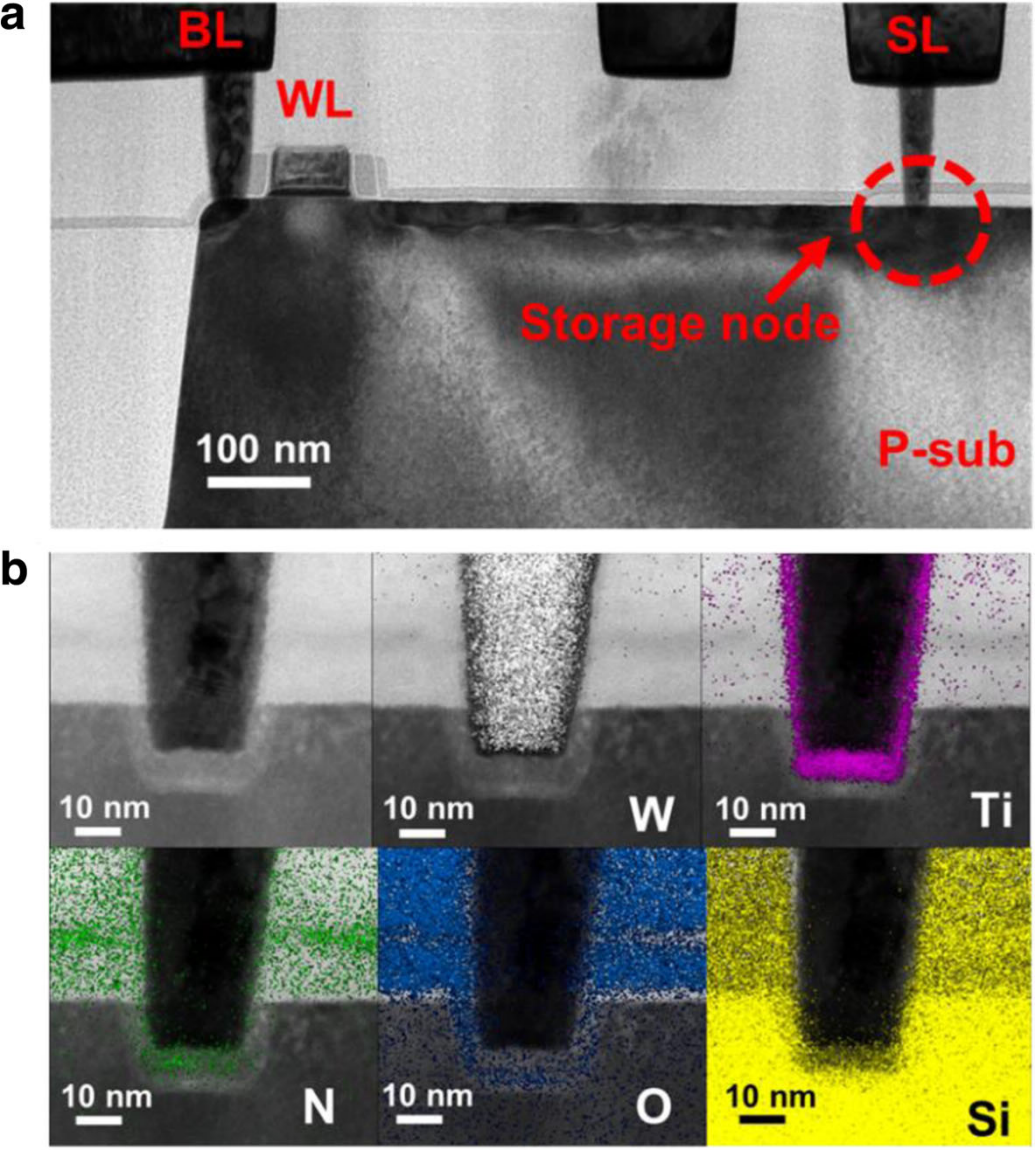
**a** Cross-sectional TEM image of 1T1R CRRAM structure. **b** Composition mapping of CRRAM. The resistive switching film is composed of TiN/TiON/SiO_2_ sandwiched between the top tungsten plug and bottom Si electrode

The measurement data for further statistical analysis on variability are collected from 16 × 16 contact RRAM (CRRAM) arrays which were fabricated by 28-nm CMOS logic processes, where the fabrication process of CRRAM is illustrated in Fig. 1 [21]. The resistor protection oxide (RPO) layer and interlayer dielectric (ILD) are first deposited after the front-end process is completed with the transistors formed. To construct a functional resistive switching film, proper contact hole sizing, contact size of 30 nm × 30 nm, is performed to prevent shorting the W-plug and the n + diffusion region. Finally, the barrier layer, TiN, and tungsten plug are deposited individually. The cross-sectional TEM image of CRRAM is shown in Fig. 2a. As revealed in the picture, CRRAM is serially connected with an n-channel select transistor. A 1T1R structure is adopted to ensure proper selection in an array and prevent overshoots. Figure 2b shows the composition mapping of CRRAM. Its transition metal oxide (TMO) layer, with thickness of 9 nm, composed of TiN/TiON/SiO_2_ stacked is formed between the top tungsten and bottom silicon electrodes. After device fabrication, electrical analysis and physical model building in this study are completed by Aglient 4156C semiconductor parameter analyzer and MATLAB software platform respectively.

**Fig. 3:**
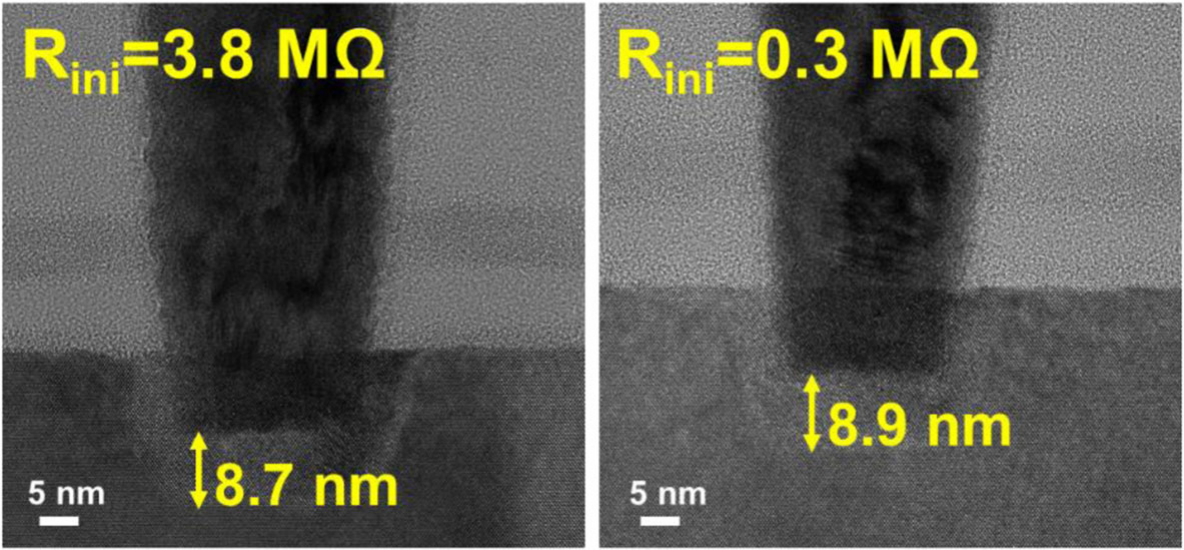
Comparison of TMO layer thickness between two CRRAM cells with great initial resistance difference. Both cells are observed with around 9-nm dielectric layer thicknesses

As reported in a previous study [22], a wide distribution of initial states is found on CRRAM array. To investigate the origin of initial state variation, thicknesses of TMO layer with different initial resistances are compared in Fig. 3 first. Data suggests no significant thickness difference between the two cells with large difference in initial resistance levels. Many studies have been reported that Vo- are generated in dielectric or RRAM film during fabrication [23–26], which implies that the difference in number and density of Vo- is expected to be responsible for the initial conductivity variations.

## Results and Discussion

### Intrinsic Vacancy Distribution Model

**Fig. 4:**
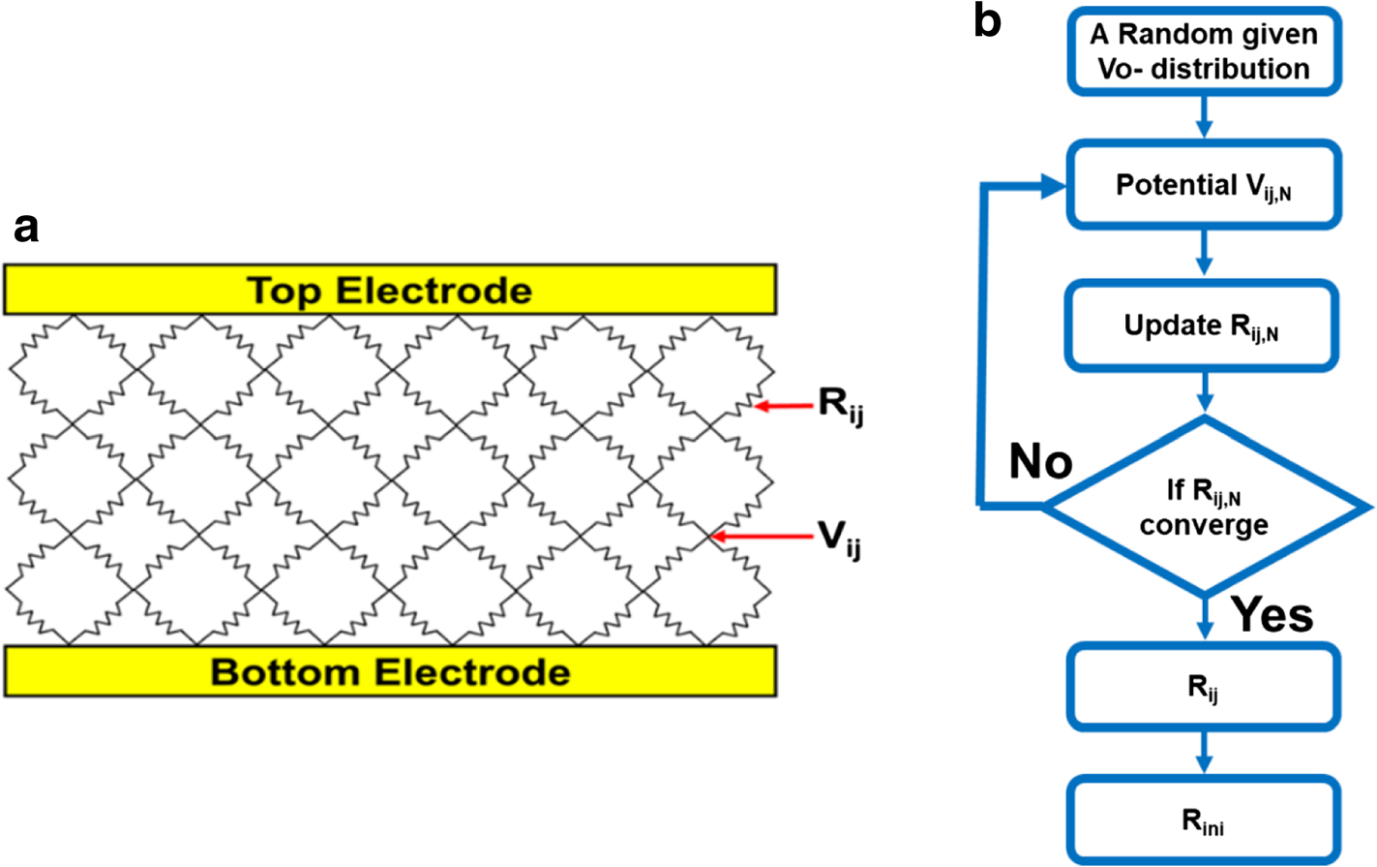
**a** Schematic of resistor network model composed by variable localized resistance of Vo-. Nodes in this network are connected to each other to simulate the interaction between Vo-. **b** Variability simulation flow of initial resistance level. Stochastic distribution of intrinsic Vo- emerge during fabrication is considered by Monte Carlo method

**Fig. 5:**
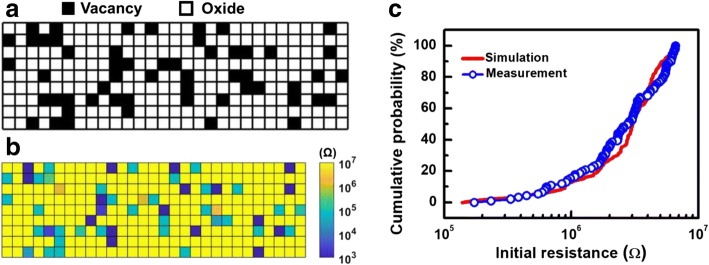
**a** Random distribution of intrinsic Vo- is initially given in RRAM film. **b** Localized resistance distribution of Vo- calculated by trap-assisted tunneling consideration. **c** *R*_ini_ distribution of fresh cells collected from CRRAM arrays agrees well with the simulation data by considering TAT conduction mechanism of preforming Vo-

**Fig. 6:**
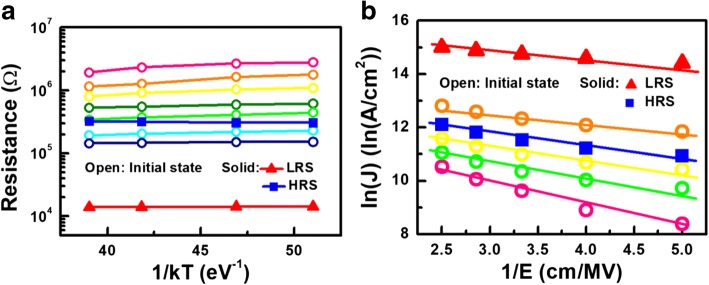
Conduction mechanism of CRRAM is determined by checking **a** temperature dependency and **b** electric field dependency. Trap-assisted tunneling followed by CRRAM is believed by two conduction characteristics, weak temperature dependency and linearly fitting between ln(J) and 1/E

To emulate the interactions between intrinsic Vo-, a resistor network model shown in Fig. 4a is established [11–14]. The resistances in each grid are calculated through a simulation flow outlined in Fig. 4b, while the corresponding physical parameters used are listed in Table 1. Based on TEM picture of CRRAM, a two-dimensional structure 30-nm width, 10 nm in thickness, is defined for describing the TMO layer, as shown in Fig. 5a. The resistance of the oxide site, *R*_oxide_, and mesh grid are determined by the material property of anatase-TiO_2_, which has been used as a resistive switching material in many studies [27–30]. Because of its tetragonal structure, the lattice constants of anatase-TiO_2_ vary with crystallographic axis. For the simplicity, mesh grids in our model are all set to be 1 nm by introducing the lattice constant in the c direction of anatase-TiO_2_ [31–33]. Furthermore, resistances for grids are also determined by referring the resistivity of anatase-TiO_2_ [34, 35]. As shown in Fig. 5a, randomly distributed Vo- are given inside the 2-D mesh initially. The temperature and electric field dependencies of CRRAM’s conduction current are summarized in Fig. 6a, b, respectively. The key characteristics of trap-assisted tunneling (TAT) current are shown by its weak-temperature effect and the linear dependency between ln(J) and 1/E [17, 36]. Using the TAT conduction model, the potential profile inside the TMO film needs to be calculated first to further obtain each localized Vo- resistance. The distribution of Vo- is expected to dominantly affect conducting current as the tunneling distance varies between oxygen vacancies. The resistance of Vo-, *R*_ij_, is then calculated by Eq. 1, which considers the probabilities of Vo- presence at the site and adopts the TAT model, for computing the tunneling probability between vacancy states.

1 $$ {R}_{\mathrm{ij},N}=\frac{R_{\mathrm{oxide}}}{\alpha\ {C}_{\mathrm{Vo}-}^{\kern0.75em \beta }\ \exp \left(\frac{\phi }{d}\right)} $$

**Table 1:** Simulation parameter for imitating the behavior of trap-assisted tunneling and Vo- generation process of forming operation

Parameter	Illustration	Value
*R*_ij_	Localized resistance of Vo- site	
*V*_ij_	Potential	
*R*_oxide_	Localized resistance of oxide site	18 MΩ [34, 35]
*N*	Iteration time	
*E*	Electric field	
*ϕ*	Electric potential difference	
*d*	Tunneling distance	
*C*_Vo-_	Vo- concentration	
*R*_ini_	Initial resistance state	
*P*_ij_	Probability of Vo- generation	
*P*_g_	Threshold switching probability	
*R*_forming_	Resistance after forming operation	
*V*_f_	Forming voltage	
*α*	Fitting parameter	1660
*β*	Fitting parameter	1.3
*γ*	Fitting parameter

Each *R*_ij,*N*_ is updated in each iteration until the result converges eventually. As the final *R*_ij_ distribution is obtained, as illustrated in Fig. 5b, the overall resistance, *R*_ini_, of a fresh cell can also be projected subsequently, as shown in Fig. 5c. As can be seen in Fig. 5c, the variation of simulated *R*_ini_ distribution obtained by proposed simulation flow considering stochastic distribution and concentration of intrinsic Vo- agree fair well with the distribution of the *R*_ini_ measured on CRRAM arrays. Therefore, randomly distributed intrinsic Vo- in TMO layers, creating multiple tunneling paths, contribute to the widely spread initial resistance found in preforming CRRAM arrays.

### Analysis of Non-uniform Forming Process

**Fig. 7:**
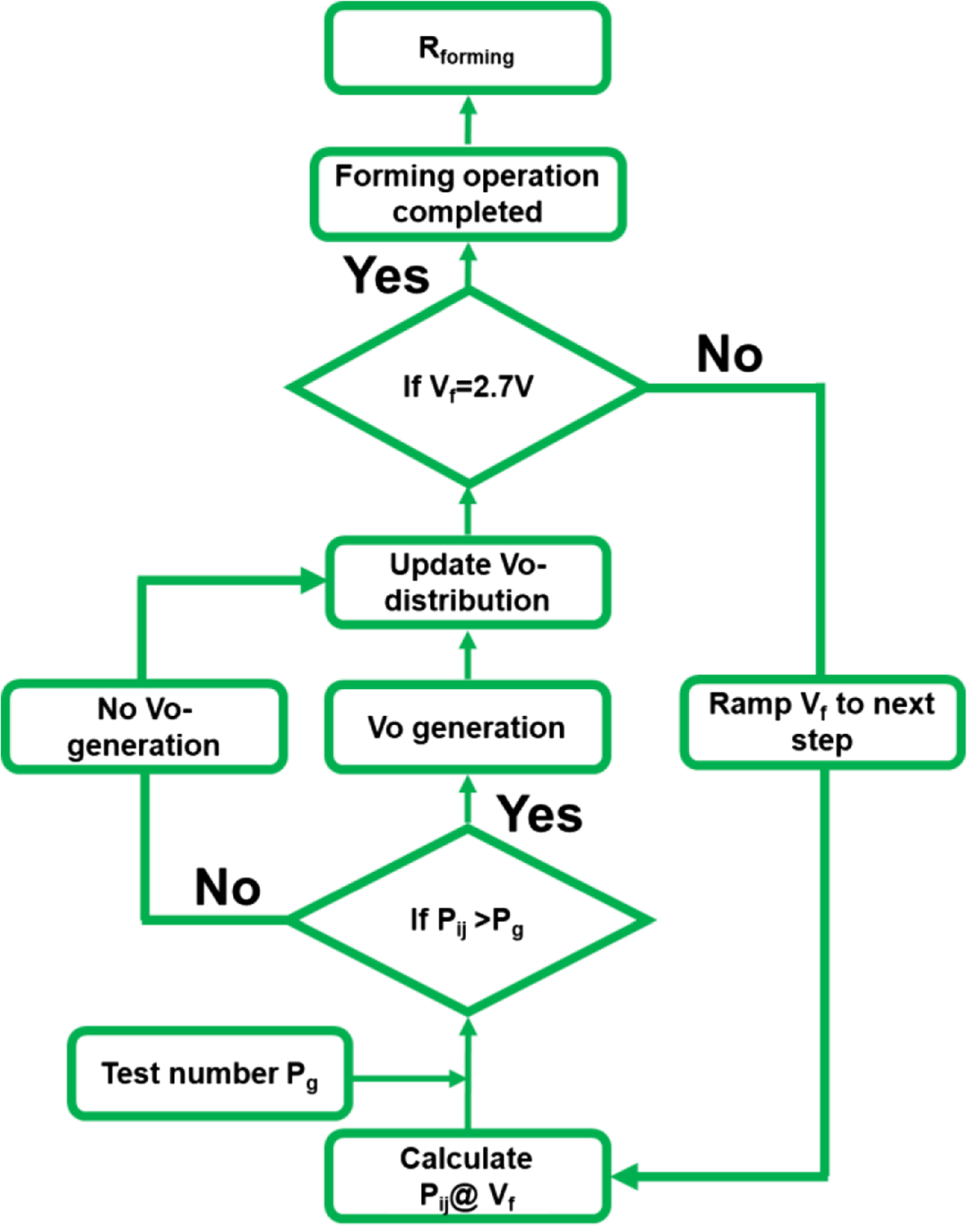
Simulation flow of a forming process based on the thermal chemical model, by assuming the dielectric failure time with electric field dependency of exp.(−E)

**Fig. 8:**
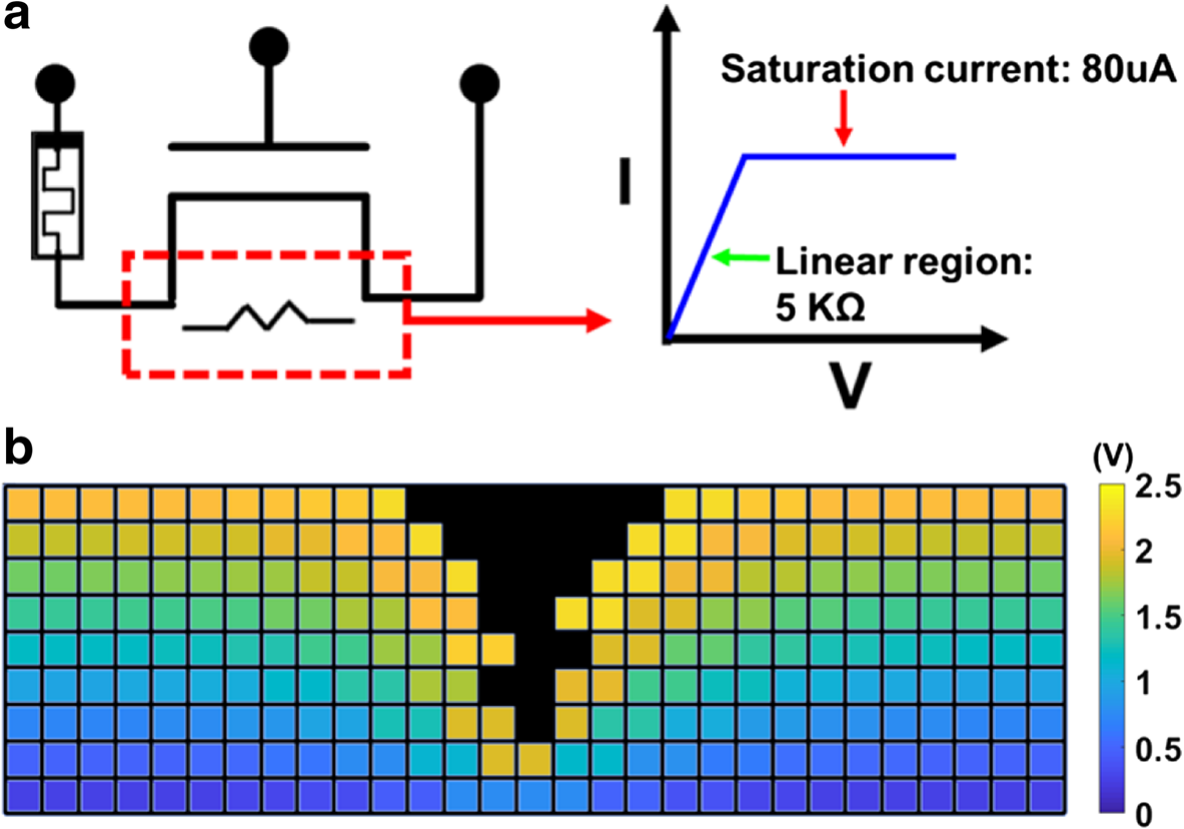
**a** Forming operation is simulated by a CRRAM serially connected with an ideal transistor. **b** Non-uniform electric potential distribution, resulting from pre-existing Vo-, induces localized field and accelerates the generation of new defects

After modeling causes attributed to the cell-to-cell variation in the fresh state, forming operation, initializing the resistive switching characteristics, is analyzed. The simulation flow of forming operation under DC sweep mode is shown in Fig. 7 [18–20]. As depicted in Fig. 8a, a cell is connected to a select transistor in series with a channel resistance of approximately 5 KΩ in linear region and a saturation current of around 80 μA. As a result of the low forming voltage, the conduction and stress mechanisms of dielectric in low electric field regime must be considered. Based on the thermal chemical model proposed in previous studies, accurate prediction of dielectric failure has been demonstrated [37–40]. Theoretical breakdown behavior of TiO_2_ simulated by the thermal chemical model [41] has shown similar characteristics as that observed in CRRAM. Therefore, the Vo- generation rate is obtained based on the thermal chemical model here [42–44]. As suggested by the thermal chemical model, the grid points beside Vo- are defined as a weak spot in the vicinity surrounding the defects. The presence of Vo- also induces localized enhanced field, shown in Fig. 8b, and accelerates the generation process of Vo- [45]. Considering the time to dielectric breakdown process in the thermal chemical model with a field dependency of exp.(−E), the probability of Vo- generation *P*_ij_ is calculated by the following equation [42].

2 $$ {P}_{\mathrm{ij}}=\gamma\ \exp \left(\mathrm{E}\right)\ \left\{\begin{array}{c}\kern1.75em \upgamma =0,\mathrm{if}\ \mathrm{site}\ \mathrm{is}\ \mathrm{not}\ \mathrm{weak}\ \mathrm{spot}\\ {}\upgamma =1,\mathrm{if}\ \mathrm{site}\ \mathrm{is}\ \mathrm{weak}\ \mathrm{spot}\end{array}\right. $$

**Fig. 9:**
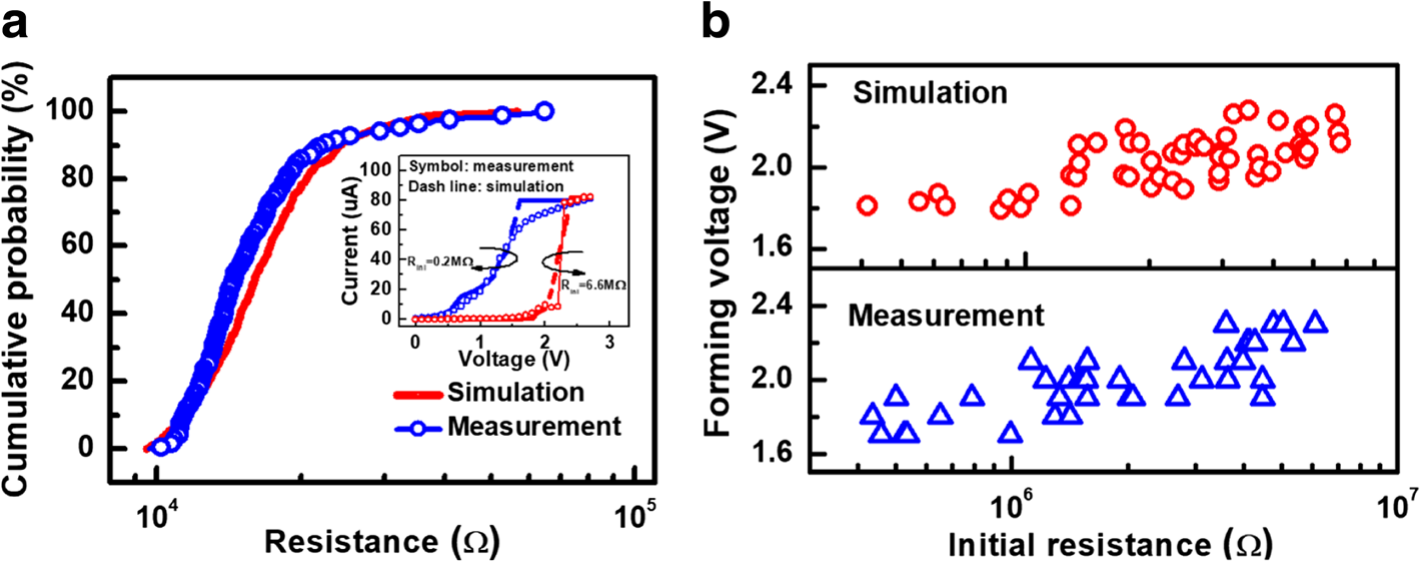
**a** Simulated resistance distribution of forming operation agrees well with measurement result. **b** Positive correlations between initial resistance and forming voltage are found in both measured and simulated data due to more weak points and higher electric field strength produced by preforming. Vo-

A critical level, *P*_g_, and a criterion, *P*_ij_ > *P*_g_, are defined for whether a new Vo- is generated. A ramping process is applied to update new Vo- distribution at each iteration until forming voltage reaches 2.7 V. Finally, with a randomly distributed intrinsic Vo-, the low resistance level *R*_forming_ after forming operation can be obtained. Based on the above model, the simulated *R*_forming_ distribution projected a wide variation, as shown in Fig. 9a, and the calculated *I-V* characteristics agree well with measured data. Furthermore, the correlation between forming characteristics and initial states is also investigated. Higher concentration and localized distributed Vo- accelerate the forming process. Therefore, positive correlation between forming voltage and *R*_ini_ are found in both simulation results and measured data, as shown in Fig. 9b.

**Fig. 10:**
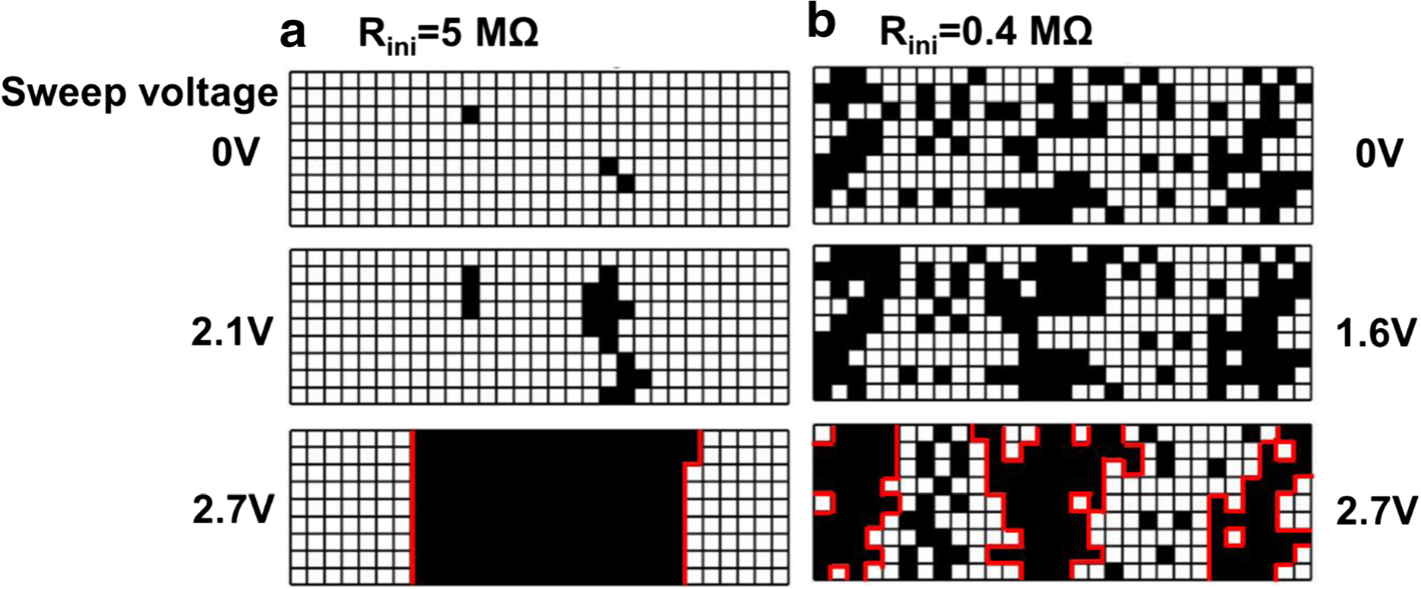
Progress of CF in cell with **a** high initial resistance and **b** low initial resistance. Higher intrinsic Vo- concentration in the TMO layer results in Vo- randomly generation at weak spots. These Vo- also connect to each other to form dendritic paths

**Fig. 11:**
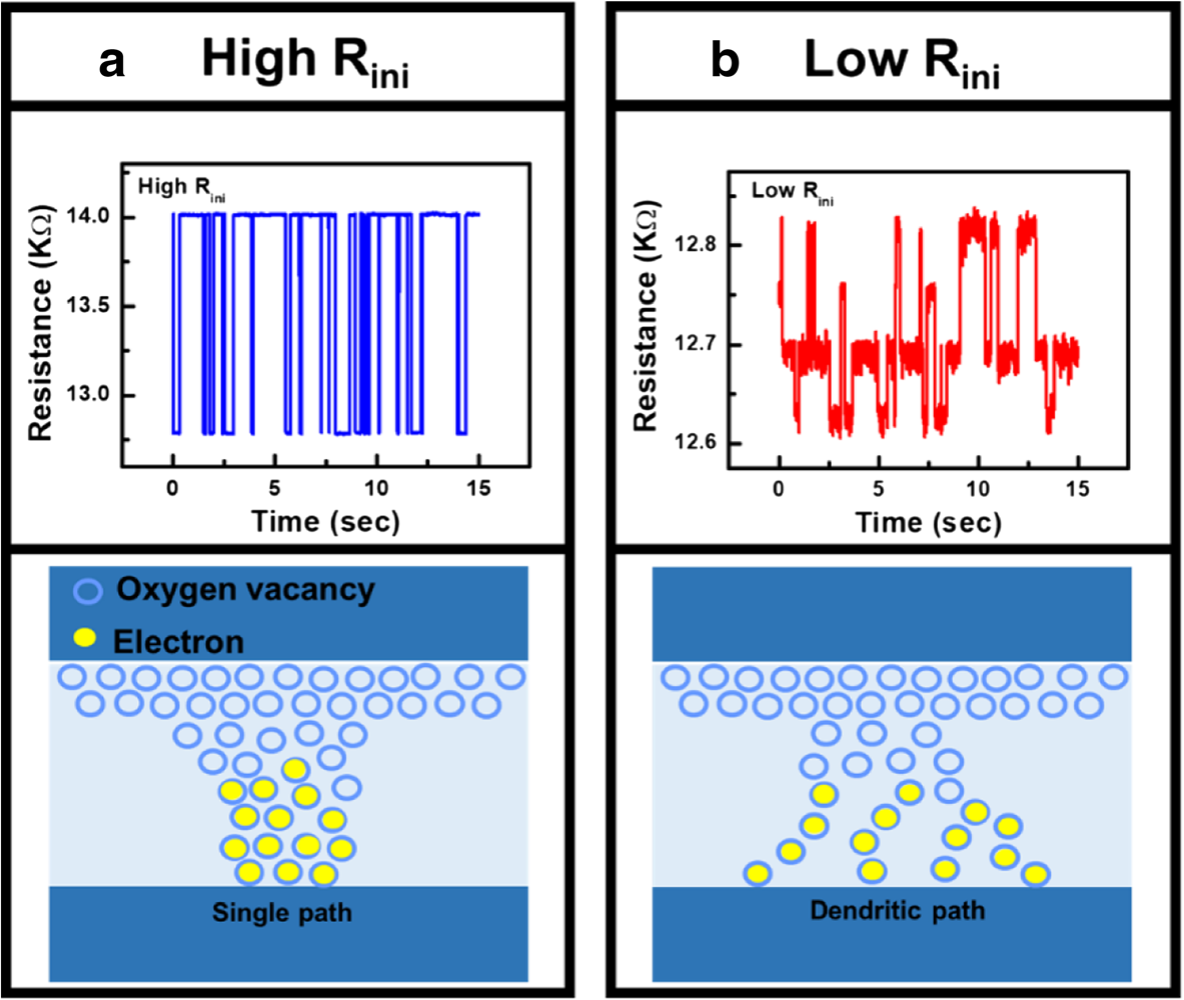
The topographies of CF in cell with **a** high initial resistance and **b** low initial resistance are analyzed by its corresponding RTN data. Occurrence of multiple resistance fluctuation in cells with low initial resistance and more intrinsic Vo- verifies the existence of dendritic CFs in TMO layer

**Fig. 12:**
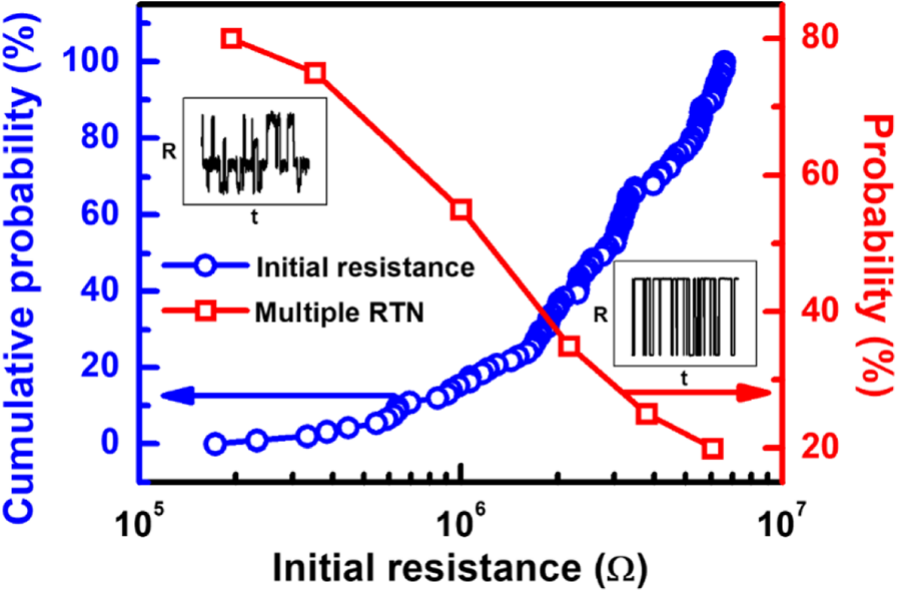
The correlation between the initial resistance level and RTN level on CRAM cells is summarized. Higher probability of bi-level resistance fluctuation is expected to occur for cells with one dominant conductive path, which correlated strongly with cells of high *R*_ini_

**Fig. 13:**
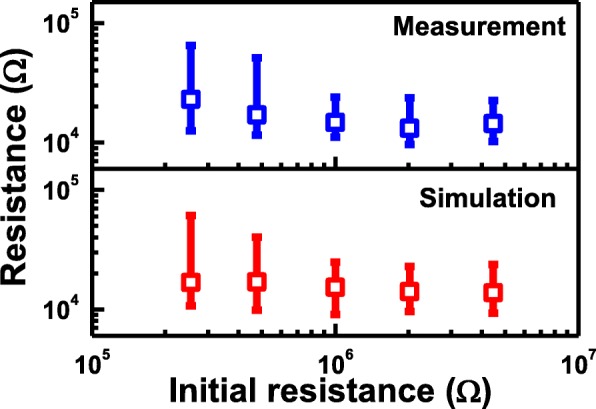
Analysis of resistance level variation after forming operation is examined through both simulation and measurement. Higher variation induced by dendritic CF generation is found in cells with low initial resistance

Moreover, Vo- generated in forming operation induces conductive path and result in a change of CF in cells, where the evolution of CF during forming process is depicted in Fig. 10. For cells with high *R*_ini_, there are fewer intrinsic Vo- and less weak spots, as illustrated in Fig. 10a. After the forming operation, a single conductive path is more likely to occur between the electrodes. However, growth of CF in cells with a lot of intrinsic Vo- shown in Fig. 10b tends to be more widespread; hence, dendritic CF are generated after forming. The correlation between different CF topographies and the Vo- distribution at its fresh state is also verified by measurement data. Vo- and CF in TMO layer are known to lead to distinctive random telegraph noise (RTN) during electron trapping/de-trapping process [46]. Resistance fluctuations occur if conductive path is blocked by trapped electrons, and the resistance decreases when electron de-traps. RTN analysis of CRRAM after forming is summarized in Fig. 11. Regular two-step resistance fluctuation is found in cells with high *R*_ini_, when electron trapping/detrapping takes place in a device with one dominant CF. On the other hand, multiple-level RTN is found in cells with low *R*_ini_ which is expected to obstruct the dendritic CF with more than one pathway. Statistical result of RTN is summarized in Fig. 12, by analyzing RTN measurement of more than 200 CRRAM cells. Data suggests that cells with high *R*_ini_ tend to exhibit only bi-level RTN, which more likely occurred in devices with one dominant CF [46–49]. The resistance variation after forming operation is arranged in Fig. 13. Data suggests that higher resistance variation are found in both measurement and simulation result in the cells with low *R*_ini_. As the less-confined CFs push the select transistor entering the saturation region early, a cell might not be properly formed, leading to a wider low-resistance state resistance levels.

**Fig. 14:**
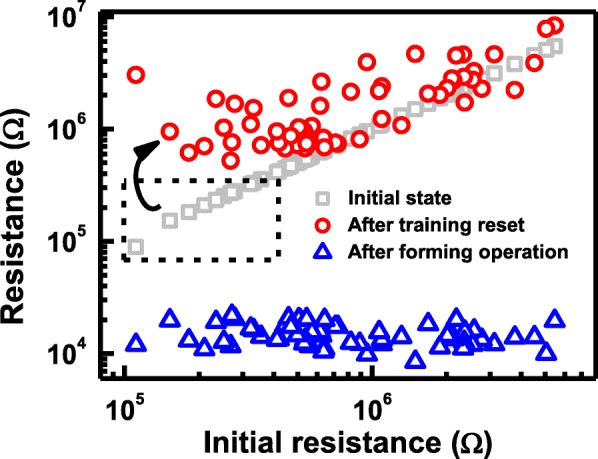
A blanket reset training operation is proposed to be applied on the CRRAM array. Resistance in cells with low *R*_ini_ is increased by annihilating intrinsic defects, but cells with high *R*_ini_ is not disturbed

To relieve forming variability caused by intrinsic Vo- in the TMO layer, a reset training operation, which sweeps SL to 1.4 V under a fixed WL voltage 2 V, is proposed to be applied blindly on whole memory cells in CRRAM array before forming. This operation is expected to annihilate pre-existing defects existing in cells with low *R*_ini_ and to ensure a better confined CF growth during the subsequent forming process. Due to low applied voltage, there is no change in cells with high *R*_ini_ after the training process. With a blanket reset training operation, the resistance of cells with low *R*_ini_, increases without disturbing the cells with high *R*_ini_, as shown in Fig. 14. Subsequently, more uniform forming characteristics can be obtained.

## Conclusions

A resistor network model considering the local field effect and trap-assisted tunneling conduction between Vo- has been successfully established. By Monte Carlo simulation, cell variability on its initial resistance as well as forming process is investigated. The variation in the fresh states of CRRAM can be successfully explained by a randomly given distribution of intrinsic Vo-. Projected resistance distribution after forming also agrees well with the measurement result by adopting the thermal chemical model. The growth of CF during forming is discussed and linked with variability observed in this process. Finally, a reset training operation is proposed to further relieve the forming variability caused by intrinsic Vo- in the TMO layer. A strong correlation between initial states and forming characteristics provide guidelines for new adaptive operations for future development of RRAM technologies.

## Abbreviations

CF: Conductive filament
CRRAM: Contact resistive random access memory
C_Vo-_: Vo- concentration
d: Tunneling distance
E: Electric field
ILD: Interlayer dielectric
*N*: Iteration time
*P*_g_: Threshold switching probability
*P*_ij_: Probability of Vo- generation
*R*_forming_: Resistance after forming operation
*R*_ij_: Localized resistance of Vo- site
*R*_ini_: Initial resistance state
*R*_oxide_: Localized resistance of oxide site
RPO: Resistor protection oxide
RRAM: Resistive random access memory
RTN: Random telegraph noise
TAT: Trap-assisted tunneling
TMO: Transition metal oxide
*V*_f_: Forming voltage
*V*_ij_: Potential
Vo-: Oxygen vacancy
*α*: Fitting parameter
*β*: Fitting parameter
*γ*: Fitting parameter
*ϕ*: Electric potential difference
